# Assessment of Changes in Visits and Antibiotic Prescribing During the Agency for Healthcare Research and Quality Safety Program for Improving Antibiotic Use and the COVID-19 Pandemic

**DOI:** 10.1001/jamanetworkopen.2022.20512

**Published:** 2022-07-06

**Authors:** Sara C. Keller, Tania M. Caballero, Pranita D. Tamma, Melissa A. Miller, Prashila Dullabh, Roy Ahn, Savyasachi V. Shah, Yue Gao, Kathleen Speck, Sara E. Cosgrove, Jeffrey A. Linder

**Affiliations:** 1 Division of Infectious Diseases, Department of Medicine, Johns Hopkins University School of Medicine, Baltimore, Maryland; 2 Department of Pediatrics, Johns Hopkins University School of Medicine, Baltimore, Maryland; 3 Center for Quality Improvement and Patient Safety, Agency for Healthcare Research and Quality, Rockville, Maryland; 4 NORC at the University of Chicago, Chicago, Illinois; 5 Armstrong Institute of Patient Safety and Quality, Johns Hopkins University School of Medicine, Baltimore, Maryland; 6 Division of General Internal Medicine and Geriatrics, Northwestern University Feinberg School of Medicine, Chicago, Illinois

## Abstract

**Importance:**

The Agency for Healthcare Research and Quality (AHRQ) Safety Program for Improving Antibiotic Use aimed to improve antibiotic prescribing in ambulatory care practices by engaging clinicians and staff to incorporate antibiotic stewardship into practice culture, communication, and decision-making. Little is known about implementation of antibiotic stewardship in ambulatory care practices.

**Objective:**

To examine changes in visits and antibiotic prescribing during the AHRQ Safety Program.

**Design, Setting, and Participants:**

This cohort study evaluated a quality improvement intervention in ambulatory care throughout the US in 389 ambulatory care practices from December 1, 2019, to November 30, 2020.

**Exposures:**

The AHRQ Safety Program used webinars, audio presentations, educational tools, and office hours to engage stewardship leaders and clinical staff to address attitudes and cultures that challenge judicious antibiotic prescribing and incorporate best practices for the management of common infections.

**Main Outcomes and Measures:**

The primary outcome of the Safety Program was antibiotic prescriptions per 100 acute respiratory infection (ARI) visits. Data on total visits and ARI visits were also collected. The number of visits and prescribing rates from baseline (September 1, 2019) to completion of the program (November 30, 2020) were compared.

**Results:**

Of 467 practices enrolled, 389 (83%) completed the Safety Program; of these, 292 (75%) submitted complete data with 6 590 485 visits to 5483 clinicians. Participants included 82 (28%) primary care practices, 103 (35%) urgent care practices, 34 (12%) federally supported practices, 39 (13%) pediatric urgent care practices, 21 (7%) pediatric-only practices, and 14 (5%) other practice types. Visits per practice per month decreased from a mean of 1624 (95% CI, 1317-1931) at baseline to a nadir of 906 (95% CI, 702-1111) early in the COVID-19 pandemic (April 2020), and were 1797 (95% CI, 1510-2084) at the end of the program. Total antibiotic prescribing decreased from 18.2% of visits at baseline to 9.5% at completion of the program (−8.7%; 95% CI, −9.9% to −7.6%). Acute respiratory infection visits per practice per month decreased from baseline (n = 321) to a nadir of 76 early in the pandemic (May 2020) and gradually increased through completion of the program (n = 239). Antibiotic prescribing for ARIs decreased from 39.2% at baseline to 24.7% at completion of the program (−14.5%; 95% CI, −16.8% to −12.2%).

**Conclusions and Relevance:**

In this study of US ambulatory practices that participated in the AHRQ Safety Program, significant reductions in the rates of overall and ARI-related antibiotic prescribing were noted, despite normalization of clinic visits by completion of the program. The forthcoming AHRQ Safety Program content may have utility in ambulatory practices across the US.

## Key Points

**Question:**

Is a national antibiotic stewardship program for ambulatory care associated with a decrease in antibiotic prescribing?

**Findings:**

In this cohort study comprising members of 389 US ambulatory practices, including clinicians and staff, the Agency for Healthcare Research and Quality Safety Program for Improving Antibiotic Use Program addressed attitudes and culture that challenge judicious antibiotic prescribing and incorporated best practices for the management of common infections. Between September 2019 and November 2020, antibiotic prescribing at clinic visits decreased from 18% to 9%, and antibiotic prescribing at acute respiratory infection visits decreased from 39% to 25%.

**Meaning:**

In this study, the Safety Program appeared to provide a model for stewardship implementation in ambulatory care practices.

## Teaser

This cohort study examines antibiotic prescribing rates in ambulatory care practice during a stewardship intervention implemented through the Agency for Healthcare Research and Quality Safety Program for Improving Antibiotic Use.

## Introduction

Antibiotic stewardship (AS) programs are concerned with preventing potential harm from antibiotic overuse and have traditionally focused on acute care settings.^1^ However, most antibiotics prescribed in the US are prescribed in ambulatory care.^2,3^ Up to one-half of antibiotics prescribed in ambulatory care settings are not indicated, and antibiotic use in this setting has not declined in the past decade, underscoring the importance of implementing outpatient AS.^2,4,5,6,7^ The Centers for Disease Control and Prevention core elements of outpatient AS^8^ includes evidence-based guidance focusing on commitment, action for policy and practice, tracking and reporting, and education and expertise.

Despite national encouragement by government agencies, accreditation organizations, and specialty societies to expand AS to outpatient settings,^8,9,10,11,12^ little is known about successful implementation of AS in ambulatory care practices. In response to the need for an AS implementation framework in outpatient practices,^13^ the Agency for Healthcare Research and Quality (AHRQ) established the AHRQ Safety Program for Improving Antibiotic Use (hereinafter, the Safety Program) in ambulatory care. This 1-year program was available at no cost to primary and urgent care practices across the US. Through the development of a comprehensive toolkit, the Safety Program assisted participating practices with implementing sustainable AS.

The Safety Program approach was adapted from the Comprehensive Unit-Based Safety Program, a method to improve patient safety and quality by emphasizing health care worker teamwork and the science of safety.^14^ Earlier cohorts of the Safety Program focused on AS implementation in acute^15^ and long-term care.^16^ The objective of the present work was to describe the implementation of the Safety Program targeting outpatient practices and measure changes in antibiotic prescribing patterns in participating practices from baseline (September 1, 2019) to completion of the program (November 30, 2020). This period also largely overlapped with early stages of the COVID-19 pandemic.

## Methods

### Program Development and Enrollment

We performed a cohort study evaluating a quality improvement intervention in a national collaborative of ambulatory practices. The ambulatory care Safety Program was conducted December 1, 2019, to November 30, 2020, across the US. The Safety Program team included the investigators, with input from a technical panel comprising experts in AS, primary care, urgent care, implementation research, patient safety, and patient advocacy. Primary care, urgent care, student health, community-based health centers, and specialty medical practices providing primary care were recruited through strategies such as listservs; social media; outreach to relevant professional societies, state health departments, local medical societies, and area health education centers; attendance at professional conferences; federal agency engagement; quality improvement organization contacts; and insurers. Participating practices were required to use electronic health records. The project was deemed nonhuman participants research and exempt from informed consent by the Johns Hopkins School of Medicine Institutional Review Board. The study followed the Strengthening the Reporting of Observational Studies in Epidemiology (STROBE) reporting guideline.

Each practice identified a clinical and an administrative lead to oversee Safety Program implementation. All practice members were encouraged to participate, including physicians, advanced practice professionals, pharmacists, nurses, medical assistants, and front-desk staff. Each participant received unique credentials to access content. Continuing education credits were provided for physicians and advanced practice professionals, and maintenance of certification credits were available for physicians.

### Safety Program Content

The Safety Program had 3 primary goals: (1) assist practices with establishing AS infrastructure, (2) assist practice members with understanding best practices in antibiotic prescribing for common infectious conditions seen in ambulatory care, and (3) improve antibiotic prescribing teamwork, communication, and safety culture among the practice members and with patients and family members. During the 12-month Safety Program, there were 14 webinars (each repeated twice and recorded for participants, and led by S.C.K., T.M.C, and J.A.L.). Webinars were focused on the diagnosis and management of infectious diseases and used the Four Moments of Antibiotic Decision Making framework (Box).^17^ This framework encourages clinicians to answer 4 questions to determine (1) whether antibiotics are necessary, (2) what diagnostic testing is needed, (3) the safest and most effective antibiotic, and (4) ensure patients have an appropriate follow-up plan. Additional educational content included audio presentations, 1-page summary documents, patient handouts, and discussion guides (eTable 1 in the Supplement). Practices were encouraged to post commitment posters including practice member signatures or pictures to highlight the practice’s commitment to safe antibiotic prescribing.^8,18^ Participants had access to the Safety Program team via question-and-answer sessions after webinars, twice-monthly office hour sessions, and an email account. In addition, an external quality improvement expert was assigned to each practice to assist with implementing the Safety Program content through monthly calls.^19^

Box. **The Four Moments of Antibiotic Decision Making Framework**

**1. Make the Diagnosis**

Does my patient have an infection that requires antibiotics?

**2. Cultures and Empiric Therapy**

Have I ordered appropriate cultures before starting antibiotics? What empiric therapy should I initiate?

**3. Stop, Narrow, Change to Oral Antibiotics**

A day or more has passed. Can I stop antibiotics? Can I narrow therapy or change from intravenous to oral therapy?

**4. Duration**

What duration of antibiotic therapy is needed for my patient’s’ diagnosis?

Adapted from Tamma et al, 2019.^17^

The clinical and administrative AS leads were encouraged to meet with all staff monthly to accomplish the following: (1) review any updates since the last meeting and seek feedback, (2) introduce the educational topic for the month and associated material in the toolkit (eg, asymptomatic bacteriuria and urinary tract infections) and webinar dates, (3) summarize key points from the 1-page document on the monthly topic, and (4) review questions in the discussion guide with the practice (eg, What are our preferred antibiotics for treating cystitis and pyelonephritis at our clinic?). The AS leads summarized comparative, practice-level feedback data received from the Safety Program and were asked to continue the AS activities after completion of the Safety Program, and resources were provided on sustainability.

### Data Collection

Participants completed a practice assessment describing their current AS infrastructure at the beginning and the end of the Safety Program. Practices submitted monthly data from the baseline period (September 1, 2019, to November 30, 2019) through the Safety Program intervention (December 1, 2019, to November 30, 2020). Monthly data consisted of the number of visits, the number of antibiotic prescriptions written categorized by antibiotic class, the number of ARI visits, and the number of antibiotics prescribed for ARI visits. The list of oral and intramuscular antibiotics included is presented in eTable 2 in the Supplement.

Between September 2019 and February 2020, only in-person visits were included. Because many practices began offering more telemedicine visits at the onset of the COVID-19 pandemic, synchronous telemedicine visits were included from March to November 2020. Visits for ARI were identified using *International Statistical Classification of Diseases, 10th Revision* codes (*ICD-10*) (eTable 3 in the Supplement).^4^ No patient- or clinician-level information was collected.

The primary outcome of the Safety Program was antibiotic prescriptions per 100 ARI visits. The secondary outcome was antibiotic prescriptions per 100 visits. Visits per practice-month and ARI visits per practice-month were also calculated. Quarterly data reports were provided to each practice to evaluate their antibiotic use in comparison with similar practices over the course of the Safety Program (eFigure 1 in the Supplement).

To ensure accurate data collection, an instructional webinar was presented at the beginning of the Safety Program. Practices with limited experience with electronic data collection were provided instruction in extracting data from electronic health record vendors. A standardized template was developed to collect and upload data (eFigure 2 in the Supplement). The external quality improvement expert assigned for each practice reviewed their data collection techniques. Practices with data values substantially higher or lower than expected were contacted to verify submitted data.

### Statistical Analysis

Changes in practice assessment data were evaluated with the χ^2^ test. Changes in total visits, ARI visits, antibiotics per 100 visits, and antibiotics per 100 ARI visits were calculated with medians and IQRs. Additional analyses comparing antibiotic use by practice type and antibiotic class were also conducted.

Linear mixed models with random practice effects were constructed to assess changes in antibiotics per 100 visits and antibiotics per 100 ARI visits over time. Changes from baseline (September 2019) to the period of initial widespread recognition of the COVID-19 pandemic (March to May 2020), and to program end (November 2020) were estimated. Data analyses were conducted using Stata, version 16.1 (StataCorp LLC). Findings were considered significant at α < .05 with 2-sided testing.

## Results

### Safety Program Enrollment and Engagement

A total of 467 practices enrolled in the Safety Program. Of these, 389 (83%) remained in the program until its completion and 292 (75%) submitted complete data for analysis, including 6 590 485 visits to 5483 clinicians. Common reasons for withdrawal from the Safety Program included COVID-19–related competing interests (36 [46%]), data submission challenges (19 [21%]), and practice closures (14 [24%]) (eFigure 3 in the Supplement).

Participants included 82 (28%) primary care practices, 103 (35%) urgent care practices, 34 (12%) federally supported practices, 39 (13%) pediatric urgent care practices, 21 (7%) pediatric-only practices, and 14 (5%) other practice types (Table). Participating practices represented 42 states (eFigure 4 in the Supplement). Live, recorded, or transcribed webinars or audio presentations were viewed or downloaded 4349 times during the Safety Program. The most frequently viewed topic was titled Communicating With Your Patients (n = 683) (eFigure 5 in the Supplement).

**Table.** Types of Practices That Participated in the Safety Program and Submitted Sufficient Data for Analysis

Practice type	No. (%)		No. of clinicians in practices completing Safety Program, mean (SD)
	Practices that remained in Safety Program (n = 389)	Practices that submitted complete data for analysis (n = 292)	
Primary care, including pediatrics	162 (42)	103 (35)	13.3 (16.7)
Pediatric-only primary care	23 (6)	21 (7)	10.5 (10.3)
Urgent care, including pediatrics	160 (41)	141 (48)	10.5 (15.0)
Pediatric-only urgent care	40 (10)	39 (13)	9.4 (13.4)
Federally supported practices^a^	49 (13)	34 (12)	19.9 (29.6)
Other^b^	18 (5)	14 (5)	37.3 (60.6)

^a^ Included those that were primarily managed by a federal agency: Indian Health Services, the Department of Defense, and Federally Qualified Health Centers.

^b^ Included student health clinics and specialty clinics providing primary care.

### AS Implementation

The proportion of practices tracking and reporting monthly antibiotic prescriptions increased from 21% to 76% (*P* < .001). The percentage of practices that developed local guidelines for common bacterial conditions increased from 48% to 61% (*P* < .001) (eFigure 6 in the Supplement).

### Visits and Antibiotic Prescribing

**Figure 1.**
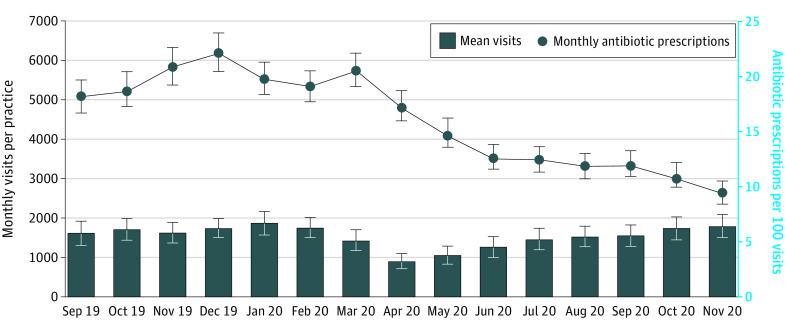
Total Visits and Antibiotic Prescriptions per 100 Visits Over Time Error bars represent 95% CIs.

Ambulatory care visits declined from baseline (mean, 1624; 95% CI, 1317-1931 visits per practice; September 2019) to a minimum at the beginning of the pandemic (mean, 906; 95% CI, 702-1111 visits per practice; April 2020) and returned to slightly more than the baseline rate at the end of the Safety Program (mean, 1797; 95% CI, 1510-2084 visits per practice; November 2020) (Figure 1). The pandemic decrease in visits was greatest for urgent care and pediatric-only practices. All practice types returned to baseline rates (eFigures 7, 8, and 9 in the Supplement). Antibiotic prescribing decreased from 18.2% of visits at baseline to 9.5% at program end (−8.7%, 95% CI, −9.9% to −7.6%) (Figure 1). There was a steep decrease in antibiotics per 100 visits between March and May 2020, from 20.6% to 14.9% (5.7%, 95% CI, 4.8%-6.6%) and antibiotics per 100 visits continued to decrease through the Safety Program to 9.5% (95%, CI, 8.4-10.5) at the completion of the intervention. The decrease in antibiotics per 100 visits was more evident for urgent care and pediatric practices (eFigures 7-9 in the Supplement) and was greatest for penicillins (eFigure 10 in the Supplement). A total of 87% of practices decreased antibiotic prescribing per 100 visits between September 2019 and November 2020 (eFigure 11 in the Supplement).

**Figure 2.**
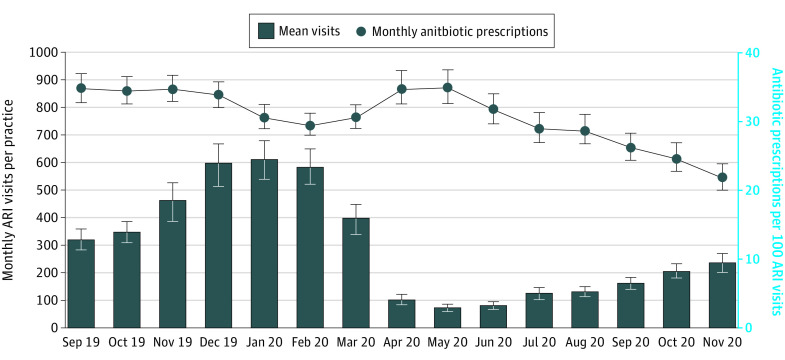
Acute Respiratory Infection (ARI) Visits and Antibiotic Prescriptions per 100 ARI Visits Over Time Error bars represent 95% CIs.

**Figure 3.**
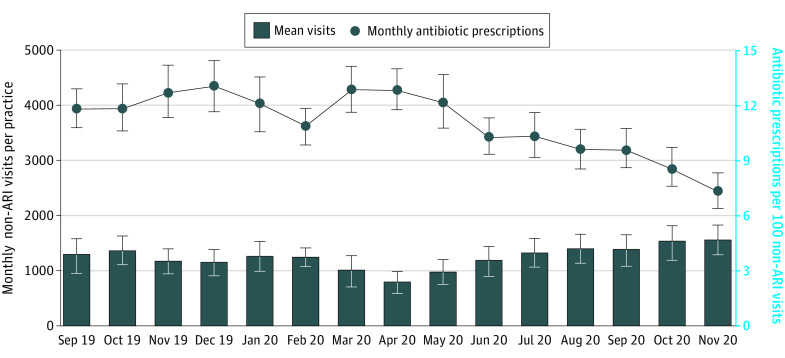
Non–Acute Respiratory Infection (ARI) Visits and Antibiotic Prescriptions per 100 Non-ARI Visits Over Time Error bars represent 95% CIs.

Visits for ARI per practice-month decreased from baseline (mean, 321; 95% CI, 282-361 ARI visits per practice; September 2019) through the beginning of the pandemic (76; 95% CI, 64-87 ARI visits per practice; May 2020) and gradually increased but remained below baseline by the end of the Safety Program (239; 95% CI, 207-270 ARI visits per practice; November 2020) (Figure 2). Antibiotic prescriptions for ARI decreased from 39.2% at baseline to 24.7% at the end of the Safety Program (−14.5%; 95% CI, −16.8% to −12.2%), although these prescriptions peaked at 39.4% (95% CI, 36.7%-42.1%) in May 2020 (Figure 2). Urgent care practices experienced the steepest decreases in antibiotics per ARI visits, from 43.7% (95% CI, 40.7%-46.7%) to 22.0% (95% CI, 19.2%-24.8%), after peaking at 48.3% (95% CI, 44.7%-51.9%) in April 2020 (eFigure 12 in the Supplement). Pediatric practices also experienced decreases in antibiotics per ARI visits, from 28.1% (95% CI, 25.1%-31.0%) to 14.7% (95% CI, 12.4%-17.0%), after peaking at 44.4% (95% CI, 39.8%-49.1%) in April 2020 (eFigure 13 in the Supplement). Similar patterns were seen in primary care practices (eFigure 14 in the Supplement). The largest decrease was observed in penicillin prescriptions (eFigure 15 in the Supplement). There was also a decrease in non-ARI visits between March and May 2020 that returned to baseline by July 2020; antibiotics per 100 non-ARI visits decreased starting in April 2020 and continued throughout the Safety Program intervention (Figure 3). After March 2020, there were increases in other respiratory diagnoses *ICD-10* codes, decreases in acute bronchitis, and very few influenza visits (eFigure 16 in the Supplement). A total of 80% of the practices decreased antibiotic prescriptions per 100 ARI visits between September 2019 and November 2020 (eFigure 17 in the Supplement).

## Discussion

In this cohort study, a year-long national program focused on AS implementation in ambulatory care practices was associated with decreases in total antibiotic prescriptions and antibiotic prescriptions for ARI visits. Antibiotic prescriptions decreased by 9 per 100 visits and antibiotic prescriptions for ARIs decreased by 15 per 100 ARI visits. The AHRQ Safety Program appears to meet an important need in ambulatory care settings where total antibiotic prescriptions are high and AS implementation strategies have been limited.^1,2,3^ Studies have reported that specific interventions may decrease ambulatory antibiotic prescribing, including educational interventions; electronic decision support; multimodal interventions; and behavioral economics nudges, including commitment posters, peer comparison feedback, and justification alerts.^20,21,22^ The decreases in antibiotic prescribing seen in the Safety Program are similar to or greater than those shown in earlier trials.^23,24,25,26^ These earlier studies were smaller and provided more practice support than the current project. We believe that the similar success, despite the intervention’s pragmatic rather than controlled nature, shows the feasibility of our approach.

The Safety Program is the first national program to provide ambulatory practices guidance in AS implementation. Evidence-based interventions for decreasing ambulatory antibiotic prescribing have been incorporated into guidance, such as the core elements of outpatient antibiotic stewardship,^8^ and associated toolkits.^27,28^ However, implementation strategies have been less well defined. We noted that approaches to improve patient safety (eg, Comprehensive Unit-Based Safety Program)^14^ by engaging clinicians and staff in communicating around antibiotic prescribing, implementing AS interventions, accessing and reviewing antibiotic prescription data, and providing evidence-based guidelines were essential components of ambulatory AS. Quality improvement experts who regularly reached out to individual practices may have also improved continued engagement.^15^ Safety Program participants appeared to have ongoing perseverance and program engagement despite significant clinical challenges during the COVID-19 pandemic.

Implementation of the Safety Program coincided with early stages of the COVID-19 pandemic, which may have affected antibiotic prescribing. Physical distancing guidelines led to a national decrease in primary care visits between March and May 2020.^29,30,31^ Similarly, as COVID-19 mitigation measures reduced the rates of visits for common respiratory viral infections,^32,33^ visits for non–COVID-19 ARIs decreased.^34^ We similarly observed decreases in total and ARI visits between March and May 2020. Two studies of US retail pharmacy data also showed a decrease in antibiotic prescribing during that period; however, this decrease was followed by an increase through December 2020.^35,36^ National pharmacy data did not reflect clinic visits, so a reduction in ambulatory care visits likely contributed to some of this national decrease in antibiotic prescribing.^29^ A single-institution study also reported that ambulatory care antibiotic prescriptions decreased at the beginning of the COVID-19 pandemic (March to May 2020), but stabilized in the subsequent months.^34^ Meanwhile, in our study, antibiotic prescribing decreased through November 2020; this decrease was in antibiotic prescribing per visit, so was not as affected by visit numbers. Furthermore, by the end of the program, practices demonstrated improvements in the infrastructure needed to sustain AS. We believe that these findings provide support for the Safety Program playing a role in a reduction in antibiotic use.

Urgent care practices had a more substantial decrease in both overall and ARI visits compared with other practice types, especially between March and May 2020. At completion of the Safety Program, urgent care ARI visits remained low while overall visits returned to near baseline, consistent with national trends.^37^ It is possible that low ARI visits but high overall visits reflect visits related to COVID-19, for which *ICD-10* codes were not captured in the data abstraction tool. Patients with COVID-19 may have been less likely to have antibiotics prescribed and more likely be seen in urgent care clinics rather than in primary care settings owing to previsit screening protocols.

Pediatric practices also had a steeper decrease in antibiotic prescriptions than adult practices. This finding is consistent with national data showing a decrease in antibiotic use to be steepest among pediatricians.^36^ Over the past decade, antibiotic prescribing overall in children has decreased while remaining stable in adults,^2,38^ possibly because pediatricians may spend a longer time with patients and reassuring the caregiver when not prescribing antibiotics.^39^

### Limitations

This study has limitations. First, despite the findings, it is difficult to distinguish the association of the COVID-19 pandemic with antibiotic prescriptions from the association of the program with antibiotic prescriptions. No control group was used, because training practices in how to perform data abstraction was a key AS intervention, which would have led to the control group being exposed to the intervention. National antibiotic prescribing data from pharmacies are not visit-based so are not directly comparable with practice-based prescribing data. We were therefore unable to compare directly with control practices. Instead, the data were qualitatively compared with published reports.^35,36,40^ Second, the Safety Program depended on practices submitting accurate data. Practices were contacted to verify out-of-range data to improve the accuracy of the information. Third, because data abstraction tools were built before the COVID-19 pandemic, data were not collected about COVID-19 diagnoses and no *ICD-10* codes were included for COVID-19. Fourth, the focus was on prescriptions, so delayed prescriptions (ie, an antibiotic prescription provided to a patient for an antibiotic-inappropriate condition with instructions to not fill the prescription unless they were still feeling poorly after several days)^8^ would have been counted.^25^ However, it is unlikely that this practice changed during the intervention because it was not discussed in the Safety Program. Fifth, called-in and handwritten prescriptions would have been missed because only electronic prescriptions were captured. However, because all practices used electronic health records, most prescriptions would have been sent electronically.

Sixth, baseline and intervention periods were only 15 months, which precluded our ability to address seasonality beyond September to November data over 2 years. Seventh, we did not collect patient-level or clinician-level antibiotic prescribing data or granular data necessary for analysis by specific diagnosis. We made these decisions to increase the feasibility and simplicity of data collection for the many practices that were not accustomed to data collection, allow for the practices to use the data in their own audit and feedback or peer comparison interventions, and allow practices to participate without individual institutional review board approval (unattainable for nonacademically affiliated practices). Eighth, although the focus was to investigate a feasible AS intervention in ambulatory practices, the practices that joined the collaborative were those that were already interested enough in AS to have garnered leadership support and identified AS leads. Our intervention may not have been as successful among practices that did not want to implement AS. Ninth, some practices dropped out of the program and others struggled to upload enough data for analysis. However, we successfully retained most practices in this pragmatic, intensive AS intervention without financial incentives or external requirements for participation, and competing demands from COVID-19, practice closures, and practice ownership changes were among the most common reasons for leaving the Safety Program.

## Conclusions

In this cohort study of US ambulatory care practices, the AHRQ Safety Program successfully launched and retained engagement in AS in a national collaboration of ambulatory care practices, despite challenges posed by the COVID-19 pandemic. There was a significant decrease in antibiotic prescribing per 100 visits and per 100 ARI visits. Addressing behavior and communication and empowering frontline staff to take part in AS was an important consideration when establishing ambulatory AS. The forthcoming AHRQ Safety Program content may have utility in ambulatory practices across the US.

## Notes

Supplement. **eTable 1.** Educational Content Provided Through the AHRQ Safety Program for Improving Antibiotic Use **eTable 2.** List of Antibiotic Prescriptions Requested From Practices **eTable 3.** Conditions and Corresponding *ICD-10* Codes for Acute Respiratory Infection Conditions **eFigure 1.** Data Feedback Report **eFigure 2.** Data Collection Form **eFigure 3.** Reasons for Withdrawal **eFigure 4.** Ambulatory Care Participants **eFigure 5.** Webinar and Audio Presentation Reach by Topic **eFigure 6.** Practice Stewardship Activities Pre-Program and Post-Program **eFigure 7.** Monthly Visits per Practice and Antibiotic Prescriptions per 100 Visits in Urgent Care Practice **eFigure 8.** Monthly Visits per Pediatric Practice and Antibiotic Prescriptions per 100 Visits **eFigure 9.** Monthly Visits per Primary Care Practice and Antibiotic Prescriptions per 100 Visits **eFigure 10.** Antibiotic Prescriptions per 100 Visits by Antibiotic Class **eFigure 11.** Antibiotic Prescriptions per 100 Visits With Variation in Change at Individual Practice Level **eFigure 12.** Monthly Acute Respiratory Infection Visits per Practice and Antibiotic Prescriptions per 100 Acute Respiratory Infection Visits in Urgent Care Practices **eFigure 13.** Monthly Acute Respiratory Infection Visits per Pediatric Practice and Antibiotic Prescriptions per 100 Acute Respiratory Infection Visits **eFigure 14.** Monthly Acute Respiratory Infection Visits per Practice and Antibiotic Prescriptions per 100 Acute Respiratory Infection Visits in Primary Care Practices **eFigure 15.** Antibiotic Prescription per 100 Acute Respiratory Infection Visits by Class **eFigure 16.** Variation in Type of ARI Visit Over Time **eFigure 17.** Antibiotic Prescribing per 100 Acute Respiratory Infection Visits With Variation in Change at Individual Practice Level
