# Supplementary material for: Assessment of Changes in Visits and Antibiotic Prescribing During the Agency for Healthcare Research and Quality Safety Program for Improving Antibiotic Use and the COVID-19 Pandemic
Source: JAMA Netw Open. 2022 Jul 6;5(7):e2220512. doi: 10.1001/jamanetworkopen.2022.20512 (PMC9260475; doi:10.1001/jamanetworkopen.2022.20512)
Supplement: Supplement. — eTable 1. Educational Content Provided Through the AHRQ Safety Program for Improving Antibiotic Use eTable 2. List of Antibiotic Prescriptions Requested From Practices eTable 3. Conditions and Corresponding ICD-10 Codes for Acute Respiratory Infection Conditions eFigure 1. Data Feedback Report eFigure 2. Data Collection Form eFigure 3. Reasons for Withdrawal eFigure 4. Ambulatory Care Participants eFigure 5. Webinar and Audio Presentation Reach by Topic eFigure 6. Practice Stewardship Activities Pre-Program and Post-Program eFigure 7. Monthly Visits per Practice and Antibiotic Prescriptions per 100 Visits in Urgent Care Practice eFigure 8. Monthly Visits per Pediatric Practice and Antibiotic Prescriptions per 100 Visits eFigure 9. Monthly Visits per Primary Care Practice and Antibiotic Prescriptions per 100 Visits eFigure 10. Antibiotic Prescriptions per 100 Visits by Antibiotic Class eFigure 11. Antibiotic Prescriptions per 100 Visits With Variation in Change at Individual Practice Level eFigure 12. Monthly Acute Respiratory Infection Visits per Practice and Antibiotic Prescriptions per 100 Acute Respiratory Infection Visits in Urgent Care Practices eFigure 13. Monthly Acute Respiratory Infection Visits per Pediatric Practice and Antibiotic Prescriptions per 100 Acute Respiratory Infection Visits eFigure 14. Monthly Acute Respiratory Infection Visits per Practice and Antibiotic Prescriptions per 100 Acute Respiratory Infection Visits in Primary Care Practices eFigure 15. Antibiotic Prescription per 100 Acute Respiratory Infection Visits by Class eFigure 16. Variation in Type of ARI Visit Over Time eFigure 17. Antibiotic Prescribing per 100 Acute Respiratory Infection Visits With Variation in Change at Individual Practice Level [file jamanetwopen-e2220512-s001.pdf]

Supplementary Online Content

Keller SC, Caballero TM, Tamma PD, et al. Assessment of changes in visits and antibiotic prescribing during the Agency for Healthcare Research and Quality Safety Program for Improving Antibiotic Use and the COVID-19 pandemic. *JAMA Netw Open*. 2022;5(7):e2220512. doi:10.1001/jamanetworkopen.2022.20512

eTable 1. Educational Content Provided Through the AHRQ Safety Program for Improving Antibiotic Use

eTable 2. List of Antibiotic Prescriptions Requested From Practices

eTable 3. Conditions and Corresponding *ICD-10* Codes for Acute Respiratory Infection Conditions

eFigure 1. Data Feedback Report

eFigure 2. Data Collection Form

eFigure 3. Reasons for Withdrawal

eFigure 4. Ambulatory Care Participants

eFigure 5. Webinar and Audio Presentation Reach by Topic

eFigure 6. Practice Stewardship Activities Pre-Program and Post-Program

eFigure 7. Monthly Visits per Practice and Antibiotic Prescriptions per 100 Visits in Urgent Care Practice

eFigure 8. Monthly Visits per Pediatric Practice and Antibiotic Prescriptions per 100 Visits

eFigure 9. Monthly Visits per Primary Care Practice and Antibiotic Prescriptions per 100 Visits

eFigure 10. Antibiotic Prescriptions per 100 Visits by Antibiotic Class

eFigure 11. Antibiotic Prescriptions per 100 Visits With Variation in Change at Individual Practice Level

eFigure 12. Monthly Acute Respiratory Infection Visits per Practice and Antibiotic Prescriptions per 100 Acute Respiratory Infection Visits in Urgent Care Practices

eFigure 13. Monthly Acute Respiratory Infection Visits per Pediatric Practice and Antibiotic Prescriptions per 100 Acute Respiratory Infection Visits

eFigure 14. Monthly Acute Respiratory Infection Visits per Practice and Antibiotic Prescriptions per 100 Acute Respiratory Infection Visits in Primary Care Practices

eFigure 15. Antibiotic Prescription per 100 Acute Respiratory Infection Visits by Class

eFigure 16. Variation in Type of ARI Visit Over Time

eFigure 17. Antibiotic Prescribing per 100 Acute Respiratory Infection Visits With Variation in Change at Individual Practice Level

This supplementary material has been provided by the authors to give readers additional information about their work.

© 2022 Keller SC et al. *JAMA Network Open.*

eTable 1: Educational content provided through the AHRQ Safety Program for Improving Antibiotic Use.

Topic	Associated Educational Material
Topics on Enhancing AS Activities	
Why Your Practice Should Focus on Antibiotic Use, and How is Your Practice Doing with Antibiotic Prescriptions?	Recorded Webinar<br>Slides and Facilitator Guide<br>Commitment Poster<br>The Four Moments of Antibiotic Decision Making Poster<br>Why Focus on Antibiotic Use Clinician One-Page Document<br>Why Focus on Antibiotic Use Discussion Guide
What Can You Learn from Your Antibiotic Prescription Data?	Recorded Webinar<br>Slides and Facilitator Guide<br>Monthly Data Collection Form
Sustaining Antibiotic Stewardship Efforts	Recorded Webinar<br>Slides and Facilitator Guide<br>Ambulatory Sustainability Work Plan
Topics on Improving Communication	
Communicating with Your Patients	Recorded Webinar<br>Slides and Facilitator Guide<br>Audio-presentation<br>"Communicating With Your Patients Around Antibiotic Decision Making" Clinician One-Page Document<br>"Communicating With Your Patients Around Antibiotic Decision Making" Discussion Guide<br>"Questions and Answers About Antibiotics" Patient and Family Handout
Developing a Consensus in Your Practice	Recorded Webinar<br>Slides and Facilitator Guide<br>"Symptomatic Treatment of Acute Respiratory Infection" Clinician One-Page Document<br>"Symptomatic Treatment of Acute Respiratory Infection" Discussion Guide
Topics on Common Infectious Diseases Syndromes	
The Never Antibiotics Diagnoses: Influenza and RSV	Recorded Webinar<br>Slides and Facilitator Guide<br>Audio-presentation<br>Influenza -– Clinician One-Page Document<br>Influenza -– Discussion Guide<br>Influenza ("The Flu") – Patient and Family Member Handout
The Never Antibiotics Diagnoses: Upper Respiratory Tract Infections	Recorded Webinar<br>Slides and Facilitator Guide<br>Audio-presentation<br>Upper Respiratory Infection ("The Common Cold") – Clinician One-Page Document<br>Upper Respiratory Infection – Discussion Guide<br>Nonspecific Upper Respiratory Infection or "Common Cold" Patient and Family Handout
The Never Antibiotics Diagnoses: Acute Bronchitis	Recorded Webinar<br>Slides and Facilitator Guide<br>Audio-presentation<br>Acute Bronchitis – Clinician One-Page Document

© 2022 Keller SC et al. *JAMA Network Open.*

	Acute Bronchitis – Discussion Guide<br>Chest Cold (Acute Bronchitis) – Patient and Family Member Handout
The Sometimes Antibiotics Diagnoses: Otitis Media	Recorded Webinar<br>Slides and Facilitator Guide<br>Audio-presentation<br>Acute Otitis Media (AOM) – Clinician One-Page Document<br>Acute Otitis Media (AOM) – Discussion Guide<br>Ear Infection (Otitis Media) – Patient and Family Member Handout
The Sometimes Antibiotics Diagnoses: Bacterial Sinusitis	Recorded Webinar<br>Slides and Facilitator Guide<br>Audio-presentation<br>Acute Sinusitis: Antibiotic Indications – Clinician One-Page Document<br>Acute Sinusitis: Antibiotic Indications – Discussion Guide<br>Viral Sinusitis – Patient and Family Member Handout
The Sometimes Antibiotics Diagnoses: Streptococcus Pharyngitis	Recorded Webinar<br>Slides and Facilitator Guide<br>Audio-presentation<br>Streptococcal Pharyngitis – Clinician One-Page Document<br>Streptococcal Pharyngitis – Discussion Guide<br>Strep Throat – Patient and Family Member Handout<br>Sore Throat (Viral Pharyngitis) – Patient and Family Member Handout
The Usually Antibiotics Diagnoses: Community-Acquired Pneumonia	Recorded Webinar<br>Slides and Facilitator Guide<br>Audio-presentation<br>Community-Acquired Pneumonia -– Clinician One-Page Document<br>Community-Acquired Pneumonia – Discussion Guide<br>Pneumonia – Patient and Family Member Handout
Management of Patients with Antibiotic Allergies	Recorded Webinar<br>Slides and Facilitator Guide<br>Audio-presentation<br>Penicillin Allergy – Clinician One-Page Document<br>Penicillin Allergy – Discussion Guide<br>Penicillin Allergy – Patient and Family Member Handout
Cellulitis and Skin Abscesses	Recorded Webinar<br>Slides and Facilitator Guide<br>Audio-presentation<br>Skin and Soft Tissue Infections -– Clinician One-Page Document<br>Skin and Soft Tissue Infections -– Discussion Guide<br>Cellulitis and Soft Tissue Infections – Patient and Family Member Handout
Asymptomatic Bacteriuria and Urinary Tract Infection	Recorded Webinar<br>Slides and Facilitator Guide<br>Audio-presentation<br>Urinary Tract Infections -– Clinician One-Page Document<br>Urinary Tract Infections -– Discussion Guide<br>Urinary Tract Infections – Patient and Family Member Handout

© 2022 Keller SC et al. *JAMA Network Open.*

eTable 2: List of antibiotic prescriptions requested from practices.

Antibiotic (Name-Brand)
AMOXICILLIN (Amoxil)
AMOXICILLIN/CLAVULANATE (Augmentin)
AMPICILLIN PO
AZITHROMYCIN (Zithromax)
CEFACLOR
CEFADROXIL (Duricef)
CEFDINIR (Omnicef)
CEFDITOREN
CEFIXIME (Suprax)
CEFPODOXIME (Vantin)
CEFPROZIL
CEFTIBUTEN
CEFUROXIME (Ceftin)
CEPHALEXIN (Keflex)
CIPROFLOXACIN (Cipro)
CLARITHROMYCIN
CLINDAMYCIN (Cleocin)
DELAFLOXACIN
DICLOXACILLIN
DOXYCYCLINE (Vibramycin)
ERYTHROMYCIN
FOSFOMYCIN (Monurol)
LEVOFLOXACIN (Levaquin)
LINCOMYCIN
LINEZOLID (Zyvox)
METRONIDAZOLE (Flagyl)
MINOCYCLINE
MOXIFLOXACIN (Avelox)
NITROFURANTOIN (Macrobid)
OFLOXACIN
OMADACYCLINE (Nuzyra)
PENICILLIN V
RIFAMPIN
SULFADIAZINE
SULFAMETHOXAZOLE/TRIMETHOPRIM (Bactrim, Septra)
TEDIZOLID
TETRACYCLINE
TRIMETHOPRIM
VANCOMYCIN PO

© 2022 Keller SC et al. *JAMA Network Open.*

eTable 3: Conditions and Corresponding ICD-10 Codes for Acute Respiratory Infection Conditions.

Condition	Corresponding ICD-10 Code(s)
Pneumonia	(J12.xx, J13, J14, J15.xxx, J16.x, J17, J18.x)
Otitis media	(H65.0x, H65.1xx, H65.9x, H66.0xx, H66.4x, H66.9x, H67.x)
Acute sinusitis	(J01.xx)
Pharyngitis	(J02.x, J03.xx)
Unspecified acute lower respiratory infection	(J22)
Acute bronchitis	(J20.x, J21.x, J40)
Influenza	(J09.x, J10.x, J11.xx) – excluding (J10.0x)
Acute laryngitis and tracheitis	(J04.xx, J05.xx)
Non-specific upper respiratory tract infection	(J00, J06.x)
Other respiratory diagnosis:	
Viral infection or unspecified site	(B34.x)
Other viral agents as the cause of disease classified elsewhere	(B97.89)
Unspecified disorder of tympanic membrane	(H73.9x)
Other specified disorders of middle ear and mastoid	(H74.8)
Unspecified disorder of middle ear and mastoid	(H74.9)
Other disorders of middle ear and mastoid in diseases classified elsewhere	(H75.xx)
Other specified disorders of nose and nasal sinuses (rhinorrhea)	(J34.89)
Other specified diseases of the upper respiratory tract	(J39.8)
Disease of the upper respiratory tract, unspecified	(J39.9)
Other respiratory disorders	(J98.xx)
Respiratory disorders in diseases classified elsewhere	(J99)
Cough	(R05)

© 2022 Keller SC et al. *JAMA Network Open.*

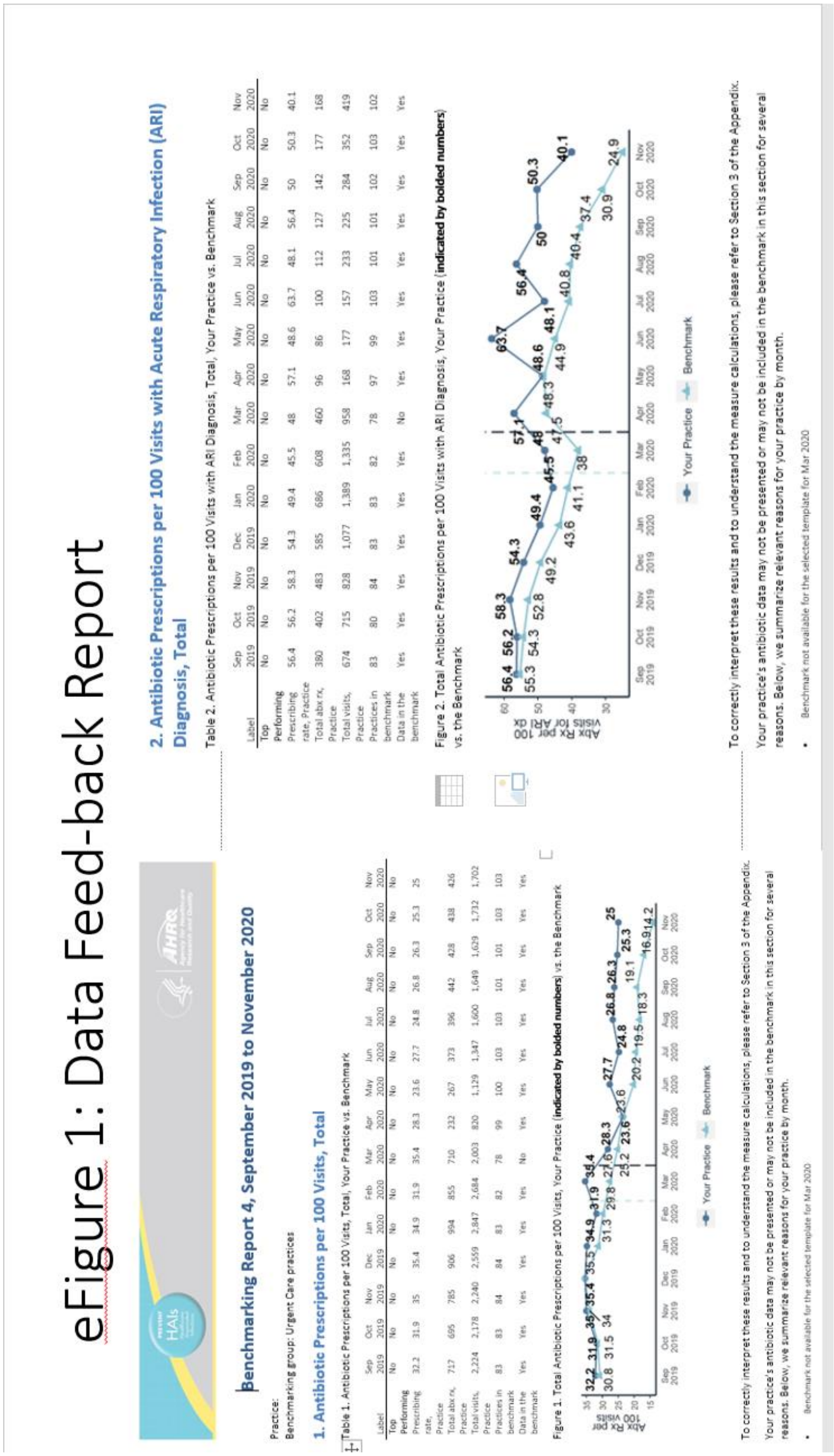

eFigure 1: Data Feed-back Report

Benchmarking Report 4, September 2019 to November 2020

Practice:

Benchmarking group: Urgent Care practices

1. Antibiotic Prescriptions per 100 Visits, Total

Table 1. Antibiotic Prescriptions per 100 Visits, Total, Your Practice vs. Benchmark

Label	Sep 2019	Oct 2019	Nov 2019	Dec 2019	Jan 2020	Feb 2020	Mar 2020	Apr 2020	May 2020	Jun 2020	Jul 2020	Aug 2020	Sep 2020	Oct 2020	Nov 2020
**Top Performing**	No	No	No	No	No	No	No	No	No	No	No	No	No	No	No
Prescribing rate, Practice	32.2	31.9	35	35.4	34.9	31.9	35.4	28.3	23.6	27.7	24.8	26.8	26.3	25.3	25
Total abx rx, Practice	717	695	785	906	994	855	710	232	267	373	396	442	428	438	426
Total visits, Practice	2,224	2,178	2,240	2,559	2,847	2,684	2,003	820	1,129	1,347	1,600	1,649	1,629	1,732	1,702
Practices in benchmark	83	83	84	84	83	82	78	99	100	103	103	101	101	103	103
Data in the benchmark	Yes	Yes	Yes	Yes	Yes	Yes	No	Yes	Yes	Yes	Yes	Yes	Yes	Yes	Yes

Figure 1. Total Antibiotic Prescriptions per 100 Visits, Your Practice (**indicated by bolded numbers**) vs. the Benchmark

To correctly interpret these results and to understand the measure calculations, please refer to Section 3 of the Appendix.

Your practice's antibiotic data may not be presented or may not be included in the benchmark in this section for several reasons. Below, we summarize relevant reasons for your practice by month.

- Benchmark not available for the selected template for Mar 2020

2. Antibiotic Prescriptions per 100 Visits with Acute Respiratory Infection (ARI) Diagnosis, Total

Table 2. Antibiotic Prescriptions per 100 Visits with ARI Diagnosis, Total, Your Practice vs. Benchmark

Label	Sep 2019	Oct 2019	Nov 2019	Dec 2019	Jan 2020	Feb 2020	Mar 2020	Apr 2020	May 2020	Jun 2020	Jul 2020	Aug 2020	Sep 2020	Oct 2020	Nov 2020
**Top Performing**	No	No	No	No	No	No	No	No	No	No	No	No	No	No	No
Prescribing rate, Practice	56.4	56.2	58.3	54.3	49.4	45.5	48	57.1	48.6	63.7	48.1	56.4	50	50.3	40.1
Total abx rx, Practice	380	402	483	585	686	608	460	96	86	100	112	127	142	177	168
Total visits, Practice	674	715	828	1,077	1,389	1,335	958	168	177	157	233	225	284	352	419
Practices in benchmark	83	80	84	83	83	82	78	97	99	103	101	101	102	103	102
Data in the benchmark	Yes	Yes	Yes	Yes	Yes	Yes	No	Yes	Yes	Yes	Yes	Yes	Yes	Yes	Yes

Figure 2. Total Antibiotic Prescriptions per 100 Visits with ARI Diagnosis, Your Practice (**indicated by bolded numbers**) vs. the Benchmark

To correctly interpret these results and to understand the measure calculations, please refer to Section 3 of the Appendix.

Your practice's antibiotic data may not be presented or may not be included in the benchmark in this section for several reasons. Below, we summarize relevant reasons for your practice by month.

- Benchmark not available for the selected template for Mar 2020

eFigure 1. Data Feedback Report

© 2022 Keller SC et al. *JAMA Network Open.*

eFigure 2. Data Collection Form

eFigure 2: Data Collection Form

Using this Form	Submission Instructions
• Please DO NOT modify the structure of the spreadsheet such as adding, deleting, or altering rows or columns<br>• Please fill in all non-colored cells on "Data Entry page 1 & 2" tabs<br>• All numbers are expected to be whole numbers.<br>• Please use the practice name that you used during website registration when entering submission information in Section 1.	• Each ambulatory care practice participating in the AHRQ Safety Program for Improving Antibiotic Use should use this spreadsheet to submit monthly data.<br>• Separate spreadsheets (data collection forms) should be submitted for each reporting month by each practice.<br>• Please speak with your Implementation Adviser regarding the timeline for the monthly data submissions or if you anticipate any difficulties collecting antibiotic usage data.

Overview of Sections

Data Entry page 1	Section 1: Submission Information	Collects information about your practice and reporting month.
	Section 2: Number of all Visits	Collects the total number of in-person and non-in-person synchronous visits.
	Section 3: All Visits (in-person and non-in-person synchronous visits) & Antibiotic Therapy Prescriptions by ARI Diagnosis	Collects number of all visits (in-person and non-in-person synchronous visits) by Acute Respiratory Infection (ARI) diagnosis, and antibiotic prescriptions during all visits (in-person and non-in-person synchronous) with ARI diagnosis.
Data Entry page 2	Section 4: Antibiotic Therapy Prescriptions for Select Antibiotics by (all Visits/ARI diagnosis & related visits)	Collects antibiotic therapy prescriptions for selected antibiotics prescribed in all visits (in-person and non-in-person synchronous), as well as those related to an ARI diagnosis.

© 2022 Keller SC et al. *JAMA Network Open.*

eFigure 3: Reasons for Withdrawal (n=78)

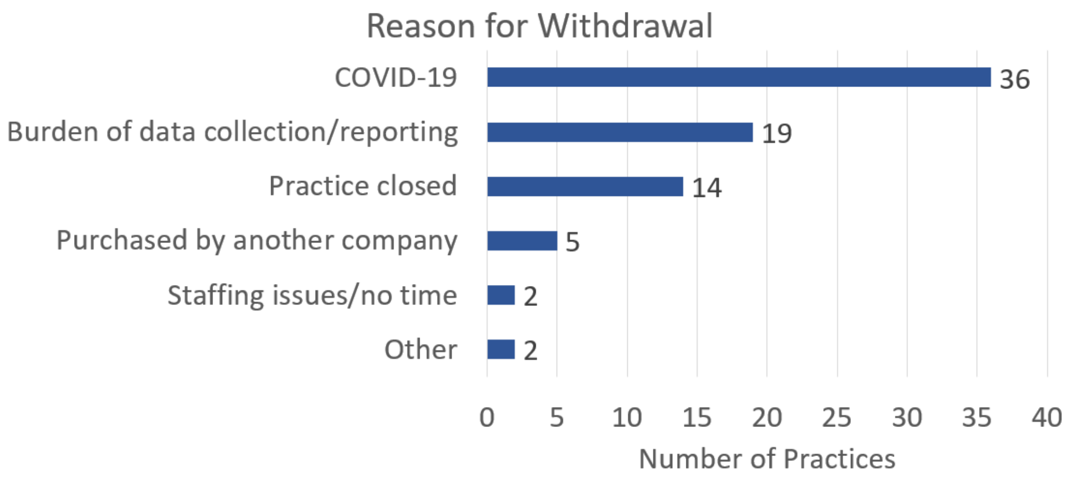

© 2022 Keller SC et al. *JAMA Network Open.*

eFigure 4: Ambulatory Care Participants (n=389)

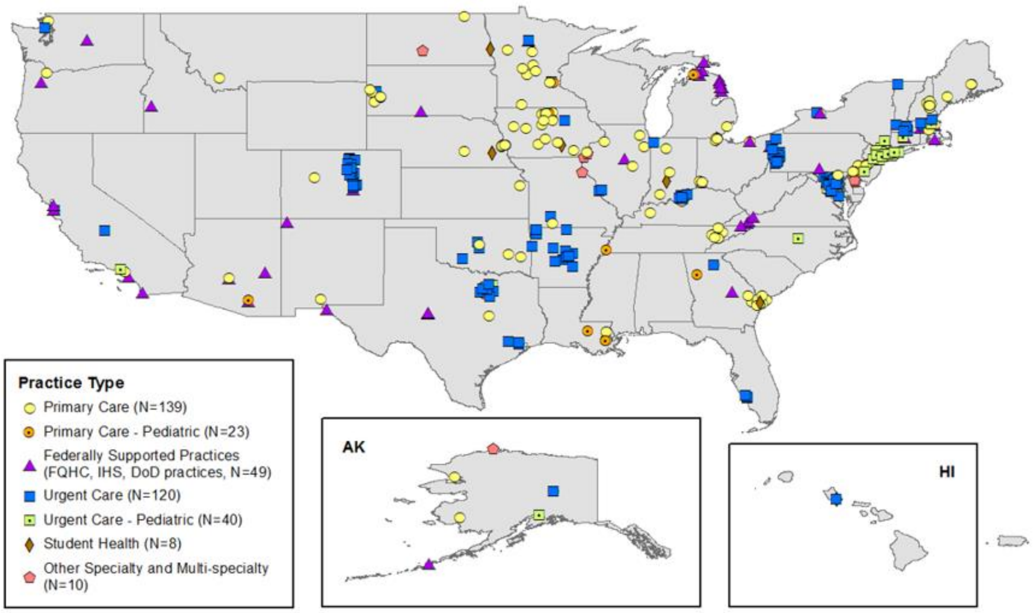

© 2022 Keller SC et al. *JAMA Network Open.*

eFigure 5. Webinar and Audio Presentation Reach by Topic

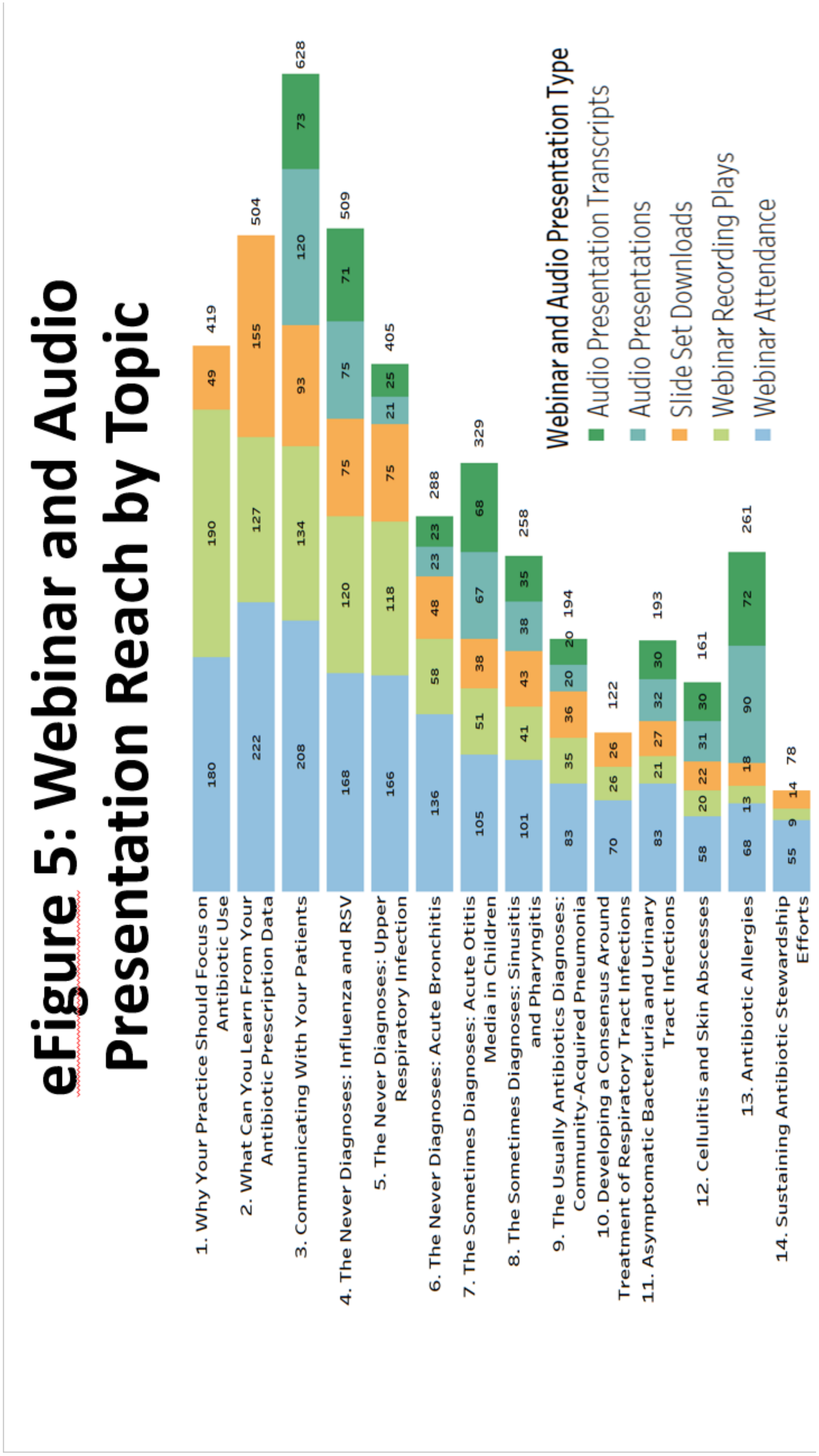

© 2022 Keller SC et al. *JAMA Network Open.*

eFigure 6: Practice stewardship activities: pre-program and post-program.

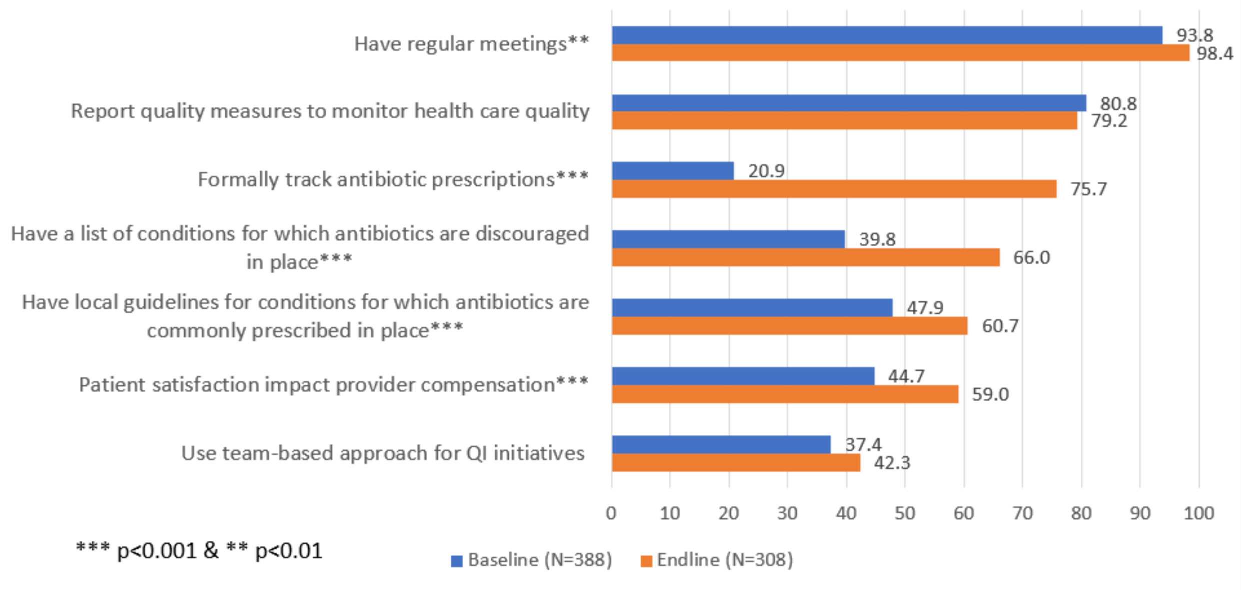

© 2022 Keller SC et al. *JAMA Network Open.*

eFigure 7. Monthly Visits per Practice and Antibiotic Prescriptions per 100 Visits in Urgent Care Practice

eFigure 7: Monthly visits per practice and antibiotic prescriptions per 100 visits in urgent care practices.

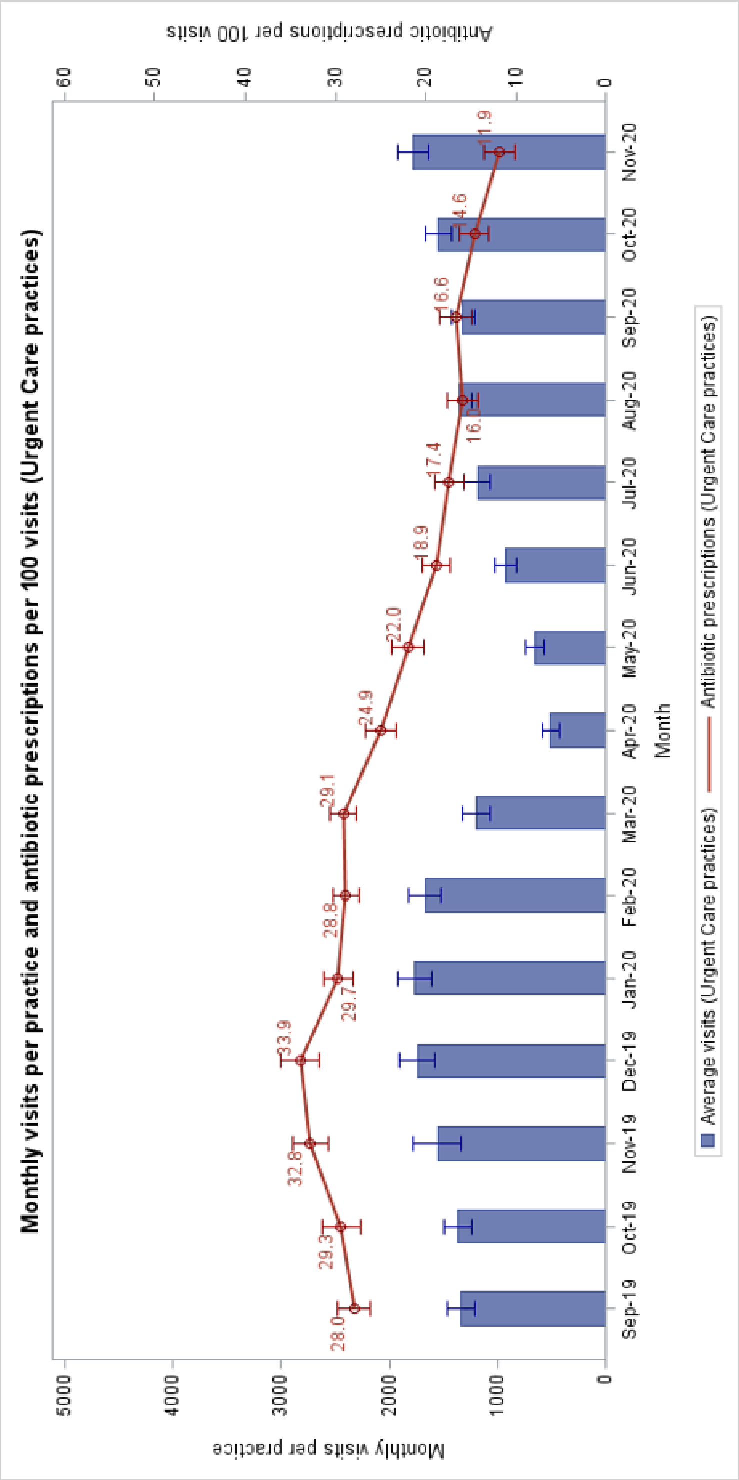

© 2022 Keller SC et al. *JAMA Network Open.*

eFigure 8: Monthly visits per pediatric practice and antibiotic prescriptions per 100 visits

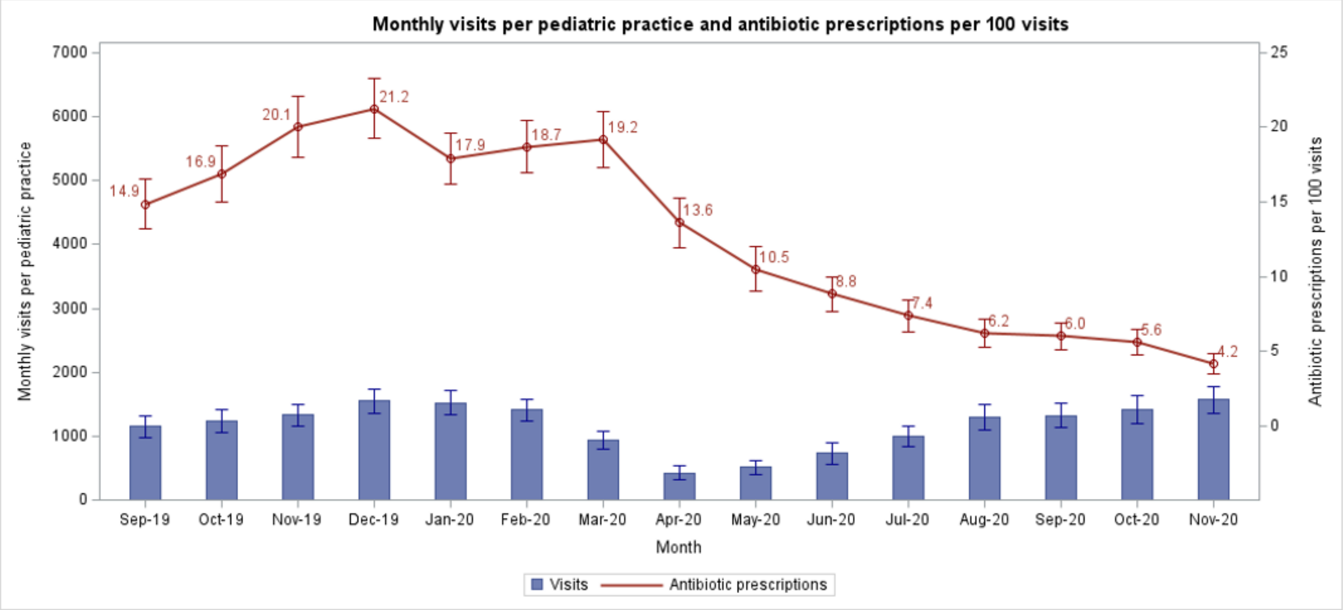

© 2022 Keller SC et al. *JAMA Network Open.*

eFigure 9: Monthly visits per primary care practice and antibiotic prescriptions per 100 visits.

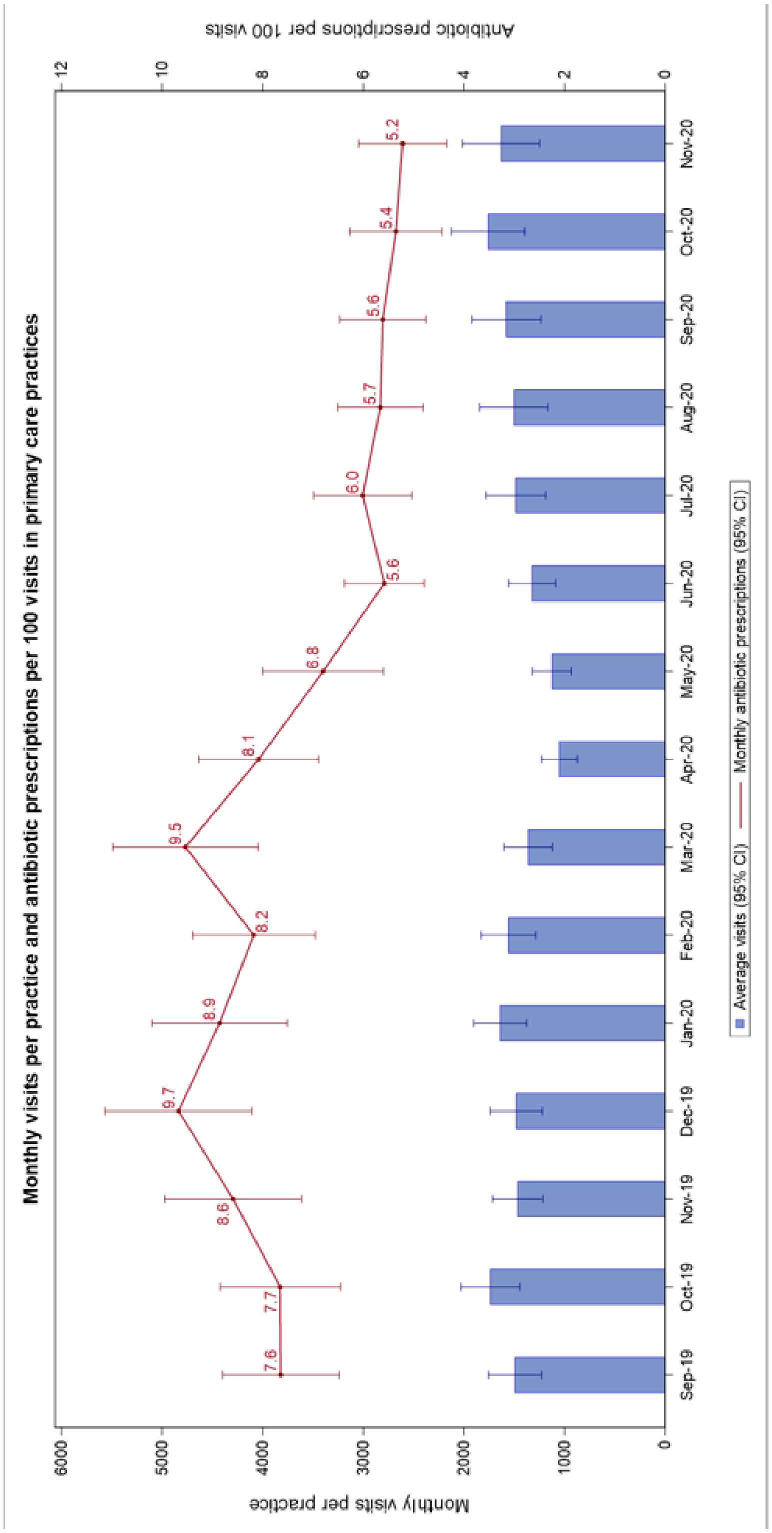

© 2022 Keller SC et al. *JAMA Network Open.*

eFigure 10. Antibiotic Prescriptions per 100 Visits by Antibiotic Class

eFigure 10: Antibiotic Prescription per 100 Visits by Antibiotic Class

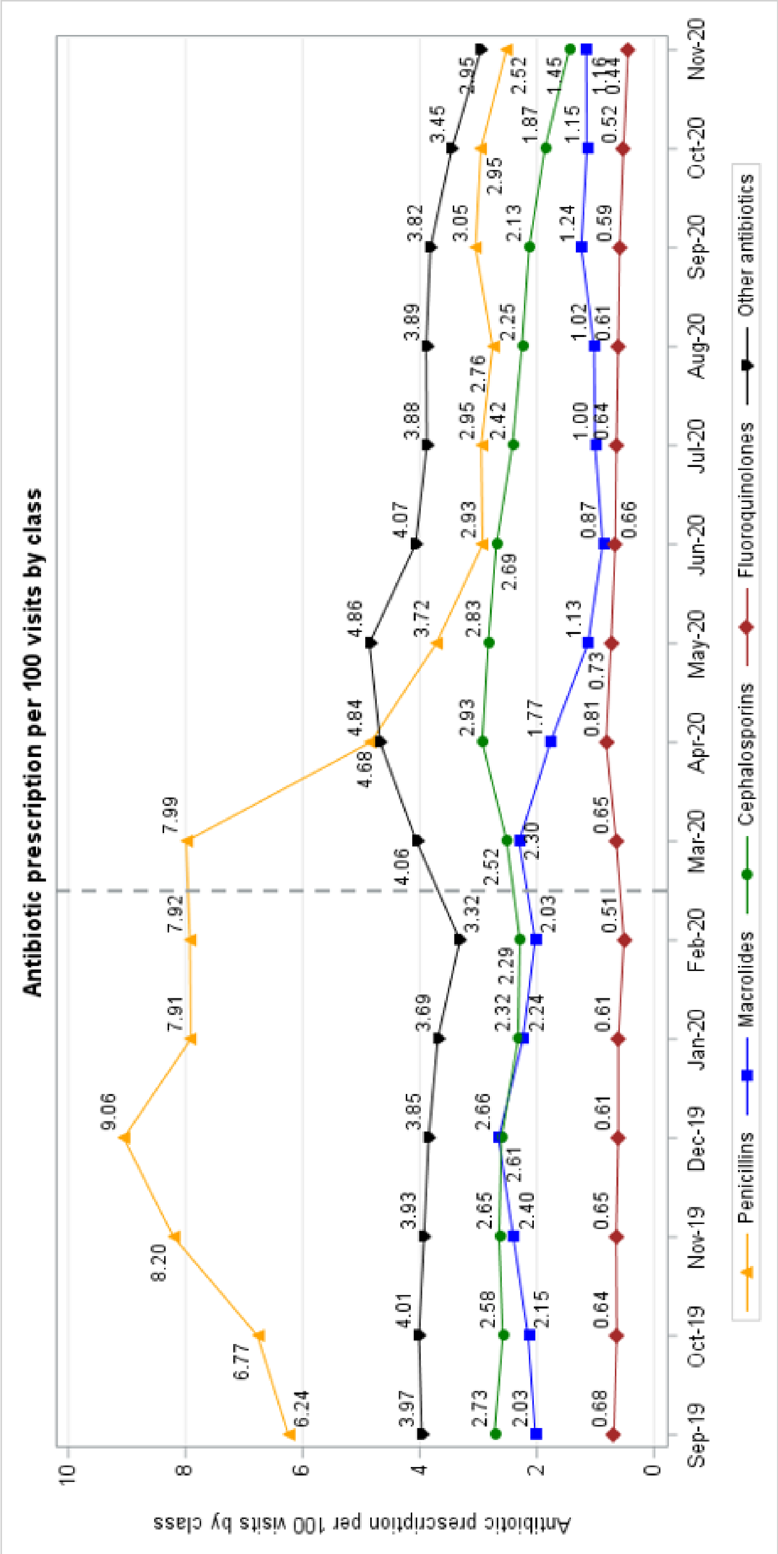

© 2022 Keller SC et al. *JAMA Network Open.*

eFigure 11. Antibiotic Prescriptions per 100 Visits With Variation in Change at Individual Practice Level

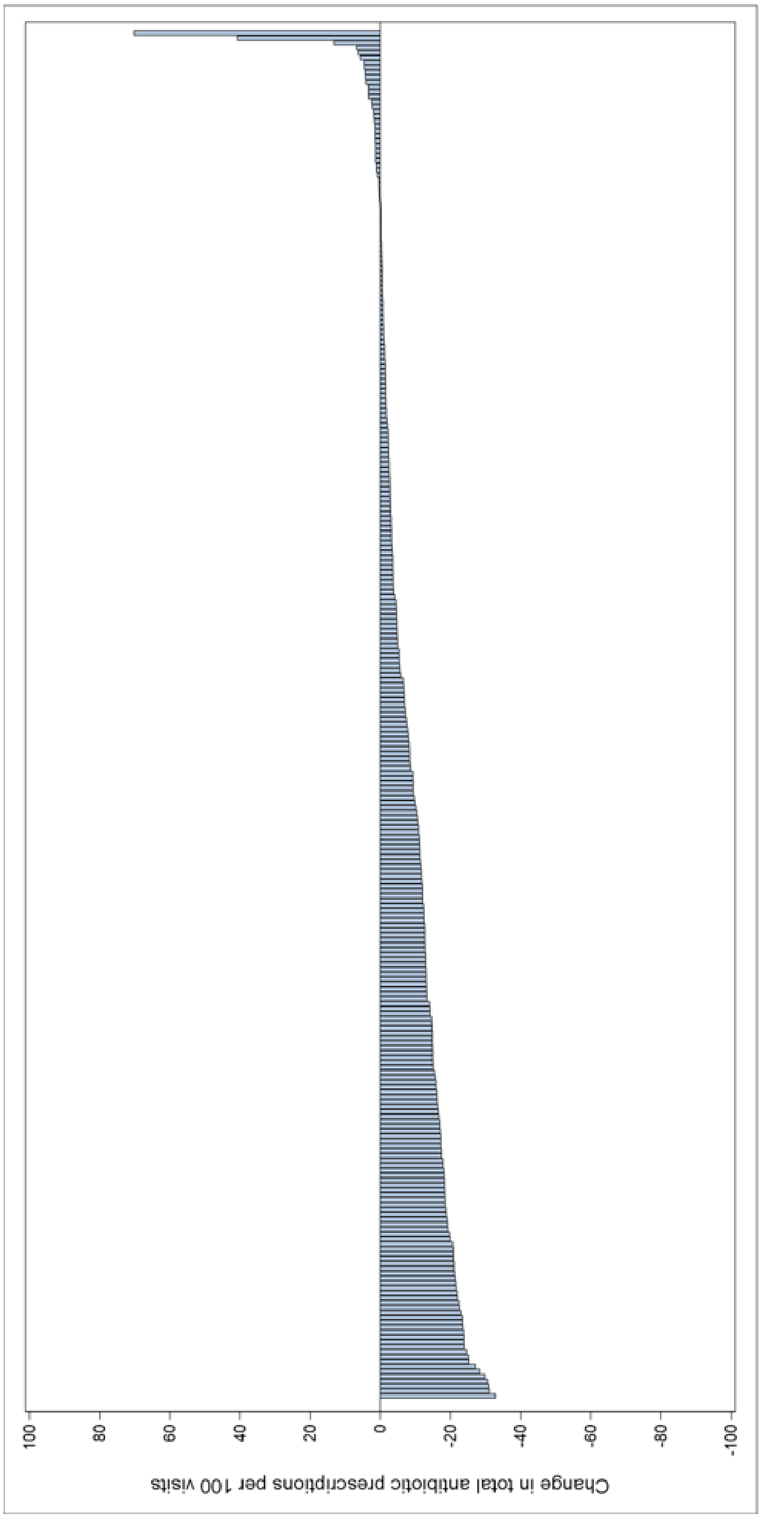

© 2022 Keller SC et al. *JAMA Network Open.*

eFigure 12: Monthly acute respiratory infection visits per practice and antibiotic prescriptions per 100 acute respiratory infection visits in urgent care practices.

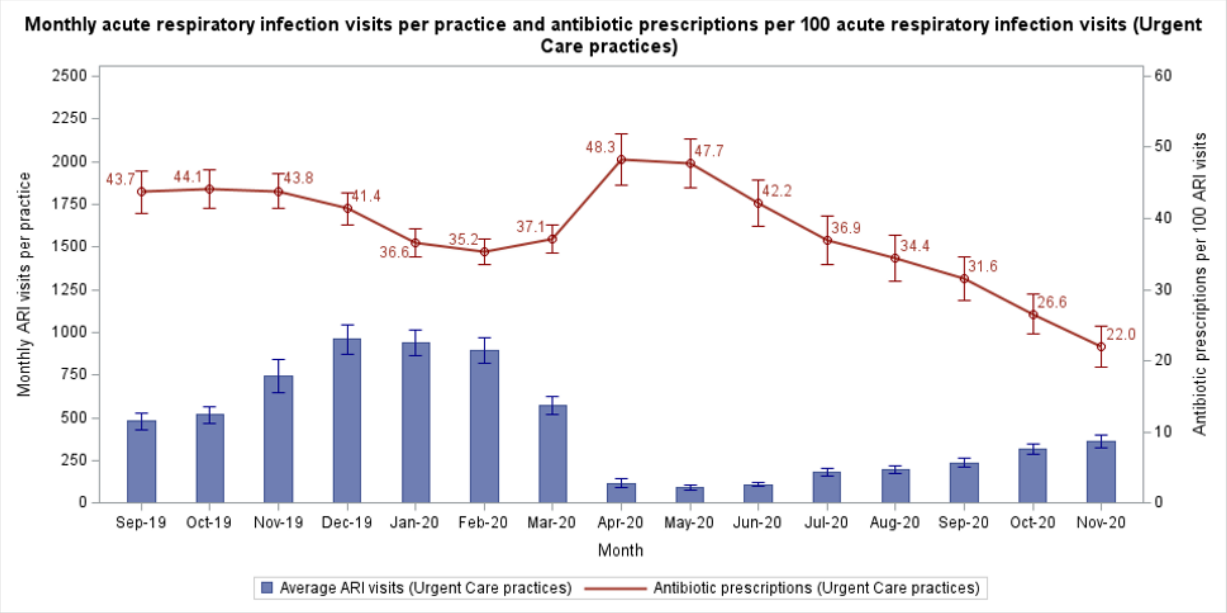

© 2022 Keller SC et al. *JAMA Network Open.*

eFigure 13: Monthly acute respiratory infection visits per pediatric practice and antibiotic prescriptions per 100 acute respiratory infection visits

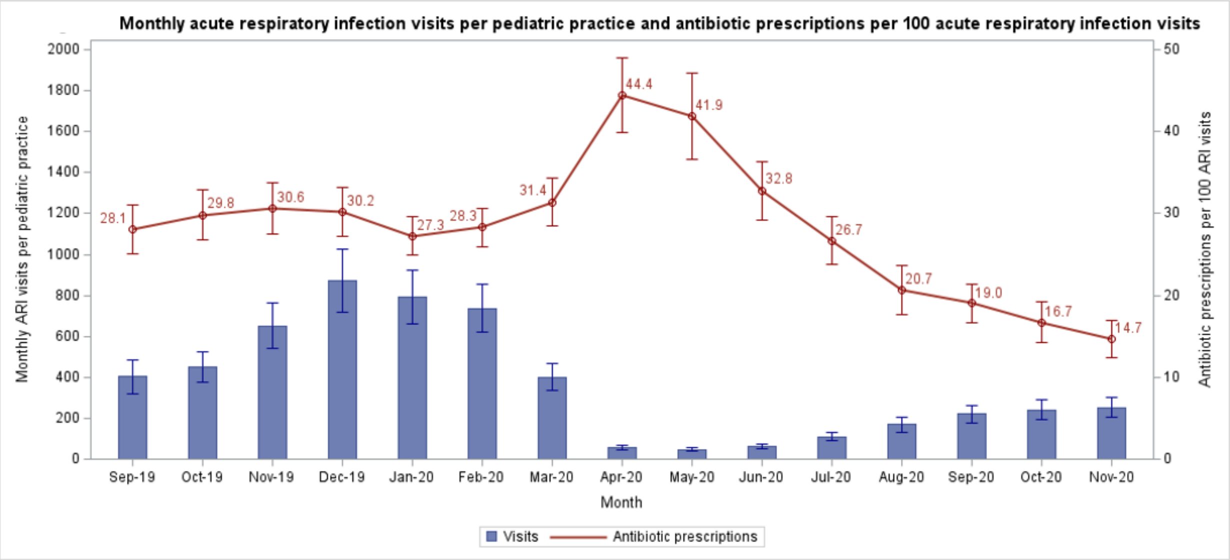

© 2022 Keller SC et al. *JAMA Network Open.*

eFigure 14: Monthly acute respiratory infection visits per practice and antibiotic prescriptions per 100 acute respiratory infection visits in primary care practices.

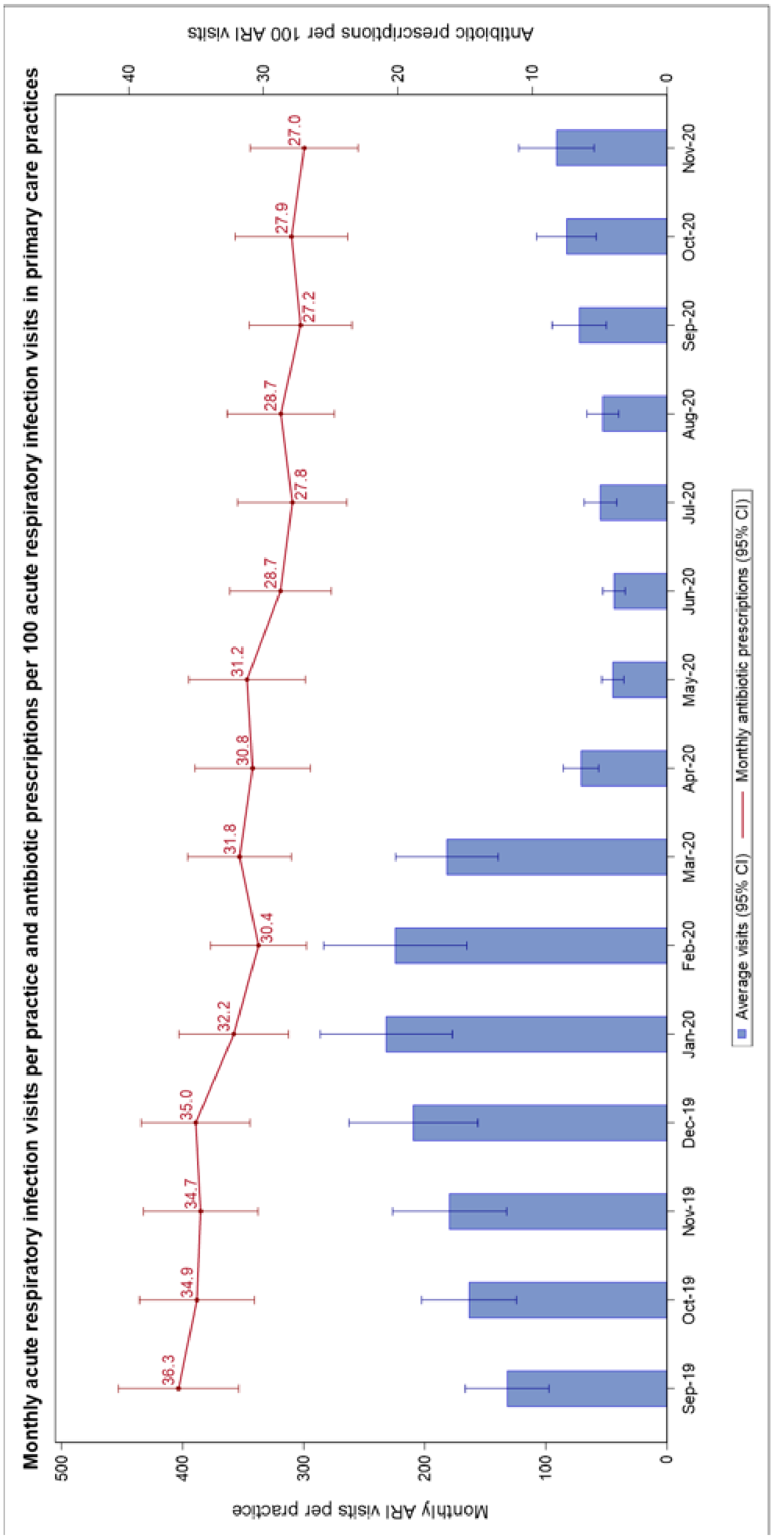

© 2022 Keller SC et al. *JAMA Network Open.*

eFigure 15. Antibiotic Prescription per 100 Acute Respiratory Infection Visits by Class

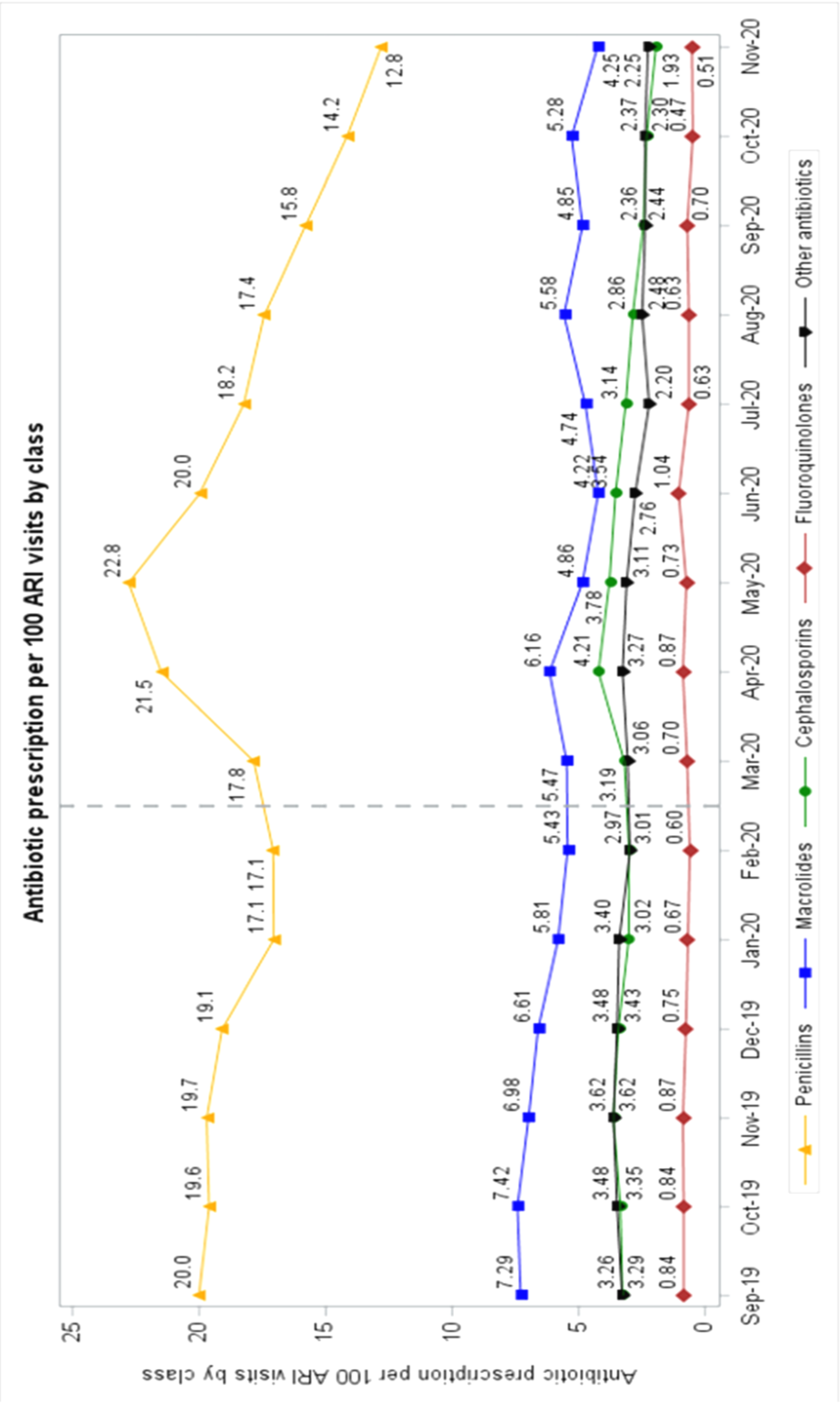

© 2022 Keller SC et al. *JAMA Network Open.*

eFigure 16: Variation in type of ARI visit over time.

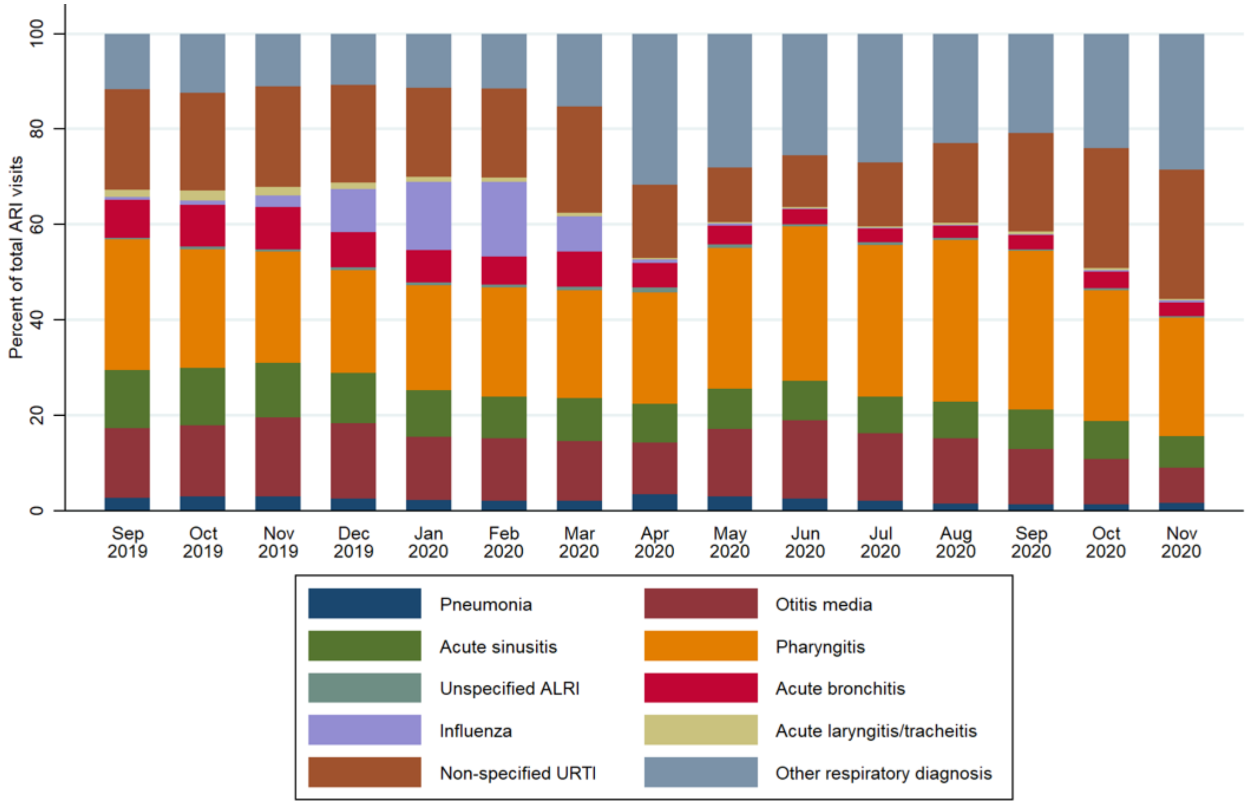

© 2022 Keller SC et al. *JAMA Network Open.*

eFigure 17: Antibiotic prescribing per 100 acute respiratory infection visits with variation in change at individual practice level.

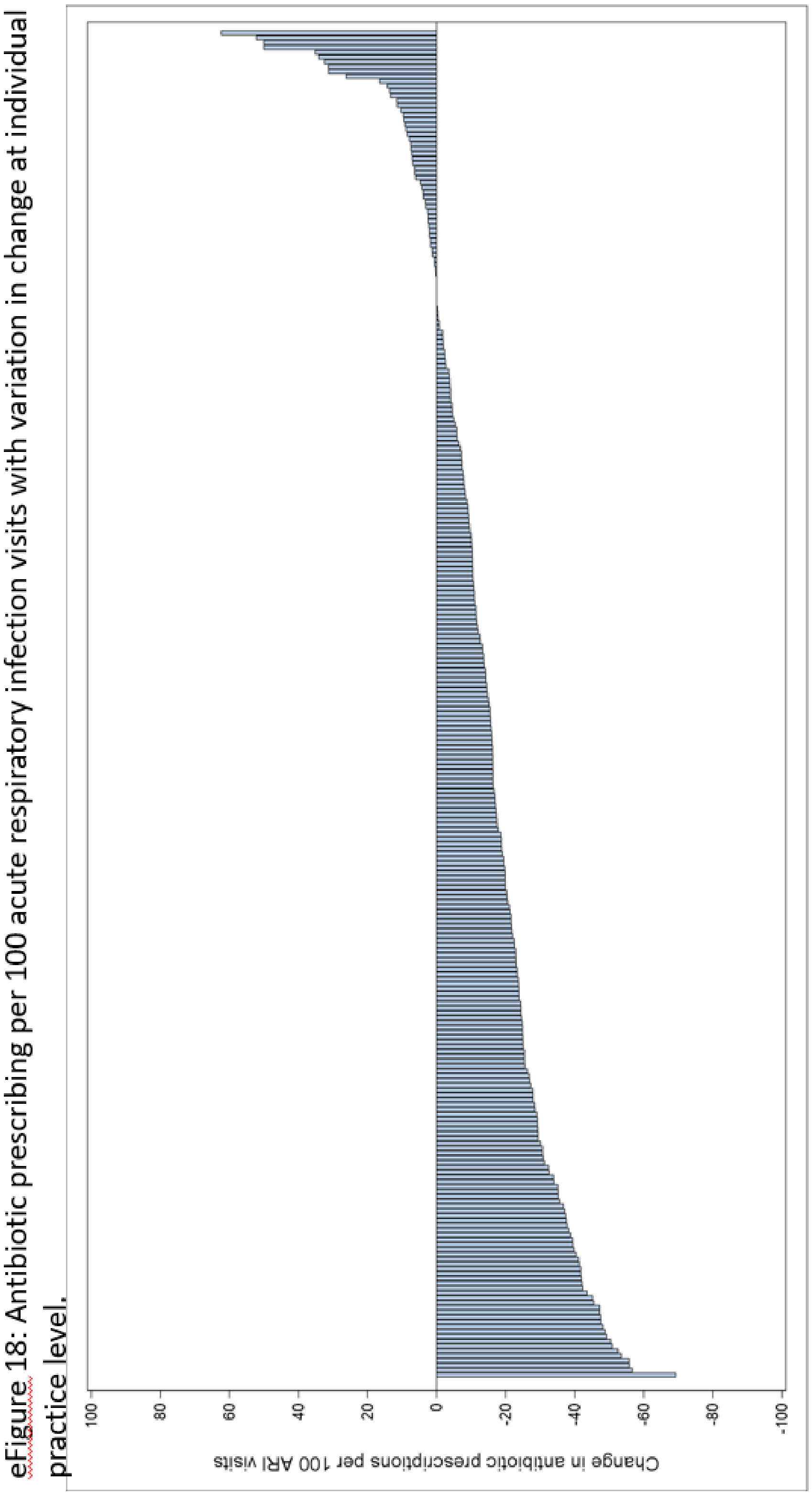

eFigure 18: Antibiotic prescribing per 100 acute respiratory infection visits with variation in change at individual practice level.

© 2022 Keller SC et al. *JAMA Network Open.*
